# Practice of Multidisciplinary Collaborative Chain Management Model in Constructing Nursing Path for Acute Trauma Treatment

**DOI:** 10.1155/2022/1342773

**Published:** 2022-07-30

**Authors:** Shuangqiong Xiang, Weiping Tang, Xiaoyuan Shang, Hongyan Ni

**Affiliations:** Department of General Surgery, General Hospital of The Yangtze River Shipping, Wuhan, Hubei 430010, China

## Abstract

Prompt and effective treatment is the key to improve the prognosis of patients with acute trauma, and nursing plays an important role. However, conventional nursing has many limitations. Some studies have pointed out that the multidisciplinary collaborative chain management model can optimize the emergency procedures, ensure the continuity of the emergency treatment process, and optimize the treatment details. This study analyzed the practice of constructing an acute trauma care pathway based on a multidisciplinary collaborative chain management model. The results showed that the application of the multidisciplinary collaborative chain management model in the construction of acute trauma treatment nursing paths can enhance the emergency efficiency and nursing treatment, prevent the occurrence of adverse events, and improve the prognosis of patients.

## 1. Introduction

The etiology of acute trauma is usually caused by the external forces such as violent blows, falling from high-rise buildings, and car accidents [1]. Due to the vigorous development of the country's transportation and construction industries, the number of acute trauma patients in China is increasing year by year [2]. Previous research data shows that, in recent years, acute trauma has replaced malignant tumors and other diseases as the main cause of death for people under the age of 45 in China [3]. Acute trauma has a golden period of rescue, during which the implementation of effective and timely treatment can improve the prognosis of patients [4] However, there are many unstable factors that affect the efficiency of patients' treatment. Nursing is an important part in the treatment of acute trauma, but there are many loopholes in the conventional emergency management [5]. For example, there is a lack of cohesion in the transfer procedures of patients, and the division of responsibilities of medical staff is not clear. Moreover, most patients with acute trauma suffer from multiple system injuries, requiring multidisciplinary consultation and treatment. However, under the conventional nursing care, each department cannot be efficiently contacted, resulting in long consultation time, delayed treatment to a certain extent, and increased treatment failure rate. Therefore, it is urgent to find an efficient nursing management mode [6]. The multidisciplinary collaboration chain management model is based on multidisciplinary cooperation and establishes an emergency treatment chain, which can not only optimize the emergency treatment process but also save the unnecessary time, ensure the continuity of the emergency treatment process, and optimize the treatment details [7, 8]. Therefore, in this study, the multidisciplinary collaboration chain management model was applied to the nursing pathway of acute trauma treatment and theeffect was observed. The results are reported as follows.

## 2. Materials and Methods

### 2.1. General Information

A total of 100 acute trauma patients who received treatment from February 2019 to February 2022 were selected. The grouping was based on the management methods. From February 2019 to August 2020, routine emergency management was adopted (*n* = 42). From September to February 2022, the management mode was multidisciplinary collaborative chain (*n* = 58). There was no significant difference in the data between the two groups (*P* > 0.05), which can be compared.

### 2.2. Inclusion Criteria

① Patients who are admitted to the emergency department by our hospital ambulance due to acute trauma; ② the time from trauma to admission is less than 3 hours; ③ patients who received treatment in our hospital all the way after admission and were discharged from the hospital; ④ patients or patients' families agree to participate in the research and have fully understood the research content.

### 2.3. Exclusion Criteria

① Patients with coagulation abnormalities such as platelet purpura; ② patients with severe underlying diseases such as malignant tumors and respiratory failure; ③ those who sign and agree to give up treatment by themselves or their family members.

### 2.4. Methods

The routine group is given routine management, specifically, sending an ambulance to the accident site to transfer the patient immediately after receiving the emergency call. The ambulance is equipped with standard drugs and equipment, and the ambulance staffing standard is a doctor, a nurse, and a dedicated ambulance driver. After reaching the accident scene, the emergency nurse cooperates with the doctor to complete the first aid measures such as fixing, bandaging, and fixing the oxygen mask and then uses a stretcher to transfer the patient to the ambulance. After arriving at the hospital, the ambulance personnel and the emergency department nurses conduct the routine vital examination and condition assessment of the patient. After basic operations such as debridement and routine intravenous infusion, the corresponding department is notified of the trauma system, such as invitingexperts from orthopaedics and other relevant departments to the emergency department for consultation and proposing measures to improve relevant examinations and treatment.

The chain group accepts a multidisciplinary collaboration chain management model, which is as follows: ① establish an emergency multidisciplinary collaborative team, which consists of acute trauma-related departments, including the emergency department, operating room, anesthesiology, imaging department, laboratory department, and the medical staff in various surgeries such as thoracic surgery, orthopedic surgery, neurosurgery, and general surgery. Refine the responsibilities of nurses and divide the nurses into four categories such as trauma nurses, circulation nurses, drug nurses, and record nurses. After the team is established, training is conducted for the respective job responsibilities of medical staff. After the training, the predrill of acute trauma treatment should be carried out for the medical staff to run in and practice with each other. ② First aid process in chain management mode: (1) prehospital first aid: after receiving the patient's call for help, one nurse from each of the four major categories and one emergency physician will promptly go to the hospital. Upon arrival to that site, the trauma nurse will assist the emergency physician in opening the wound, bandage, hemostasis, fixing the possible fracture site, and transporting the patient to an ambulance. The emergency physician should quickly conduct a physical examination and assess the patient's condition. The assessment should include the cause of the trauma, the location and type of the trauma, the organs that may be involved, the trauma index (TI), injury severity (ISS), and the Glasgow Coma Scale (GSC). The patient's vital signs are closely observed, and the nurse records the rescue process and transmits the basic information of the patient to the corresponding specialist through video, phone, and pictures. Based on the above information, the patient's basic condition is evaluated, and a treatment plan is preliminarily formulated. If necessary, the recording nurse should contact the operating room and imaging and other auxiliary departments by phone in advance to prepare surgical instruments and inspection equipment. For patients with fracture and visceral rupture due to a car accident, the trauma nurse should assist the doctor to fix the fracture position. If the patient has a broken limb, a sterile bag should be used for cryogenic refrigeration. The circulatory nurse evaluates the patient's heart rate and circulatory status, selects an appropriate respiratory support method, and administers epinephrine if necessary. The drug nurse immediately establishes venous access to replenish the blood volume, and for the patient who needs blood transfusion, blood is drawn to determine the blood type. The record nurse should contact the orthopedic trauma department and the general surgeon in advance to clarify the current blood loss, heart rate, fracture location, percussion, and auscultation results, etc., so that the orthopedic trauma surgeon can judge the severity of the fracture and formulate a surgical plan. Identify the organs that may be ruptured, confirm the estimated arrival time to the ambulance driver, and relay it to the corresponding specialist, so that the doctor can arrive in the emergency room in advance to wait. Check the imaging department according to the doctor's requirements and prepare the B-ultrasound, X-ray, and other testing equipment and the operating room. Patients with a large amount of blood loss should contact the hospital blood bank in advance to mobilize and store blood for backup, and patients with severe trauma who need surgery should contact the emergency department to open the emergency green channel. (2) Inhospital first aid: after arriving at the emergency room, improve the relevant examination immediately. Comprehensively assess the patient's condition, and make clear the location, type of trauma, whether there is hidden injury, etc. According to the evaluation results, the specialist formulates a reasonable treatment plan for treatment, and the patients who need surgery are immediately pushed into the preprepared operating room to begin the operation After the patient has been effectively treated, the nurse who is responsible for recording the emergency treatment process can inform the patient's family members of the emergency treatment process and the basic condition, treatment measures, and significance of the patient at this time, so as to do a good job in pacifying the patient's family members. The recording nurse should record the whole process of the emergency and hand it over to the chief nurse of the emergency department. The chief nurse of the emergency department will evaluate it at the next meeting, and the nurses will jointly analyze the mistakes and advantages in each process. Analyze the time spent in each process and explore whether there is an optimization plan to improve the emergency process.

### 2.5. Indicator Evaluation Criteria

① MODS: the patient has two or more organ dysfunctions [9]. ② ARDS: arterial partial pressure of oxygen/inhaled gas oxygen concentration <200 mmHg and positive end-expiratory pressure >10 cm H_2_O [10]. ③ Nursing quality: the evaluation questionnaire was used to evaluate the quality of emergency nursing care for patients with severe trauma assessed by the patient and the patient's family. The rest of the items are uniformly evaluated by the chief nurse of the department, and each item is recorded as 1–5 points, and the score is positively correlated with the quality of nursing [11].

### 2.6. Observation Indicators

Comparison of emergency triage/condition assessment/auxiliary equipment/device preparation/admission to multidisciplinary consultation/admission to receiving effective treatment time, doctor-patient disputes, misdiagnosis, missed diagnosis, MODS, ARDS, treatment success rate, hospitalization time, severe trauma, and the differences in the scores of patients' emergency nursing quality evaluation questionnaire.

### 2.7. Statistical Analysis

SPSS22.0 software was used for processing. The continuous variable data of experimental data were expressed as mean standard deviation ($\bar{x}$ ± *s*) and adopted the *t*-test. The classified variable data and descriptive analysis were expressed as (%) and adopted the *χ*^2^ test. The test level was *α* = 0.05, and *P* < 0.05 indicated that the difference was significant.

## 3. Results

### 3.1. Comparison of First Aid Efficiency between the Two Groups

The chain group was significantly lower than that of the routine group in terms of emergency triage, condition assessment, auxiliary equipment/device preparation, admission to multidisciplinary consultation, and admission to receiving effective treatment (*P* < 0.05), as shown in Table 1.

### 3.2. Comparison of the Incidence of Adverse Events between the Two Groups

The incidences of doctor-patient disputes, misdiagnosis, and missed diagnosis in the chain group were significantly lower than those in the routine group (*P* < 0.05), as shown in Table 2.

### 3.3. Comparison of Prognosis between the Two Groups

The incidence of MODS and ARDS in the chain group was lower than that in the conventional group (*P* < 0.05), and the treatment success rate was higher than that in the conventional group (*P* < 0.05) as shown in Table 3.

### 3.4. Comparison of Nursing Quality between Two Groups

The scores of system construction, first aid management, and effect evaluation of the chain group were higher than those of the conventional group (*P* < 0.05), as shown in Table 4.

## 4. Discussion

Acute trauma patients have the characteristics of critical illness and high mortality. Early identification of trauma types and conditions and the formulation of corresponding treatment plans can improve the prognosis of patients and ensure the life and health of patients. Therefore, ensuring efficient first aid and improving the quality of nursing is the focus of clinical attention [12].

The results of this study show that compared with the conventional group, the chain group has lower rates of first aid efficiency indicators and adverse time rates and higher scores on the emergency care quality evaluation questionnaire for severely traumatized patients (*P* < 0.05). This shows that this kind of management model can improve the emergency efficiency of acute trauma patients, prevent the occurrence of adverse events, and improve the quality of care, which is similar to the research results of other researchers [13]. The routine treatment of acute trauma patients is divided into four parts, and they are prehospital first aid, inhospital first aid, specialist consultation, and effective treatment. Each discipline operates independently, so the connectivity of emergency procedures is not high, and the specialists cannot grasp the patient information early and accurately to formulate preliminary treatment measures, resulting in the coherence of emergency procedures cannot being guaranteed. And, under routine management, the fuzzy division of labor among emergency nurses can lead to low first aid efficiency and affect the quality of care [14]. However, the multidisciplinary collaborative chain management model can make up for the limitations of the conventional nursing mode, and the reasons are as follows: ① multidisciplinary collaboration chain management model by establishing a multidisciplinary collaborative team, medical staff from acute trauma-related subjects are included in the team, and the preliminary assessment results of the condition are reported to the corresponding specialist through video and other forms during the first aid process, which can save consultation time and the information sharing of various departments can ensure that the specialists can grasp the patient's information at the first time, clarify the patient's condition, and prevent misdiagnosis and missed diagnosis [15, 16]. Through the preliminary diagnosis results of the specialists, the inspection equipment, operating room, and blood bank can be contacted in advance to save the preparation time of the equipment and the operating room, which can ensure the efficiency and continuity of the first aid process, simplify the rescue procedure, improve the first aid efficiency, and ensure the quality of care [17]. ② Before participating in the first aid work, the team members all carry out targeted prejob training, master the theory and time skills of job requirements, and promote the team members and nurses to run in through predrills, improve their cooperation and team cohesion, and increase their mutual coordination. ③ By clarifying the division of responsibilities of emergency nurses, performing their own duties, implementing emergency operations and responsibilities to individuals, replacing individual operations with group cooperation, and cooperating with team members, it can not only ensure the rapid progress of emergency work and improve emergency efficiency but also ensure the orderliness and pertinence in the first aid process and avoid omissions [18]. ④ Through a dedicated person to record the whole process of first aid and to appease the emotions of the patient's family in a timely manner, the patient's family can grasp the dynamics of the patient, clarify the meaning of the treatment measures, and avoid doctor-patient disputes caused by deviations and delays in information transmission [19]. ⑤ After the completion, the first aid process is reviewed to analyze the problems existing in the process, and the course of treatment is optimized, which can improve the quality of care.

Efficient emergency treatment is a key factor in ensuring acute trauma patients. Previous studies have shown that every 3-minute delay in the effective treatment of acute trauma patients can increase the risk of death by 1% [12]. With the prolongation of injury time, a large amount of blood loss and fluid loss in acute trauma patients can not only lead to hypoxia but also increase the risk of ARDS. The organ perfusion disorder caused by blood loss and fluid loss and organ damage caused by external force also increases the risk of MODS. In addition, acute trauma patients are often accompanied by the multisystem organ damage. Early understanding of the patient's condition and making a reasonable diagnosis to ensure that the patient can receive effective treatment during the golden period of treatment is an important condition to ensure the treatment effect [20]. The results of this study showed that compared with the conventional group, the chain group had a lower incidence of ARDS and MODS and a higher treatment success rate with significant differences (*P* < 0.05), which indicated that the multidisciplinary collaborative chain management model could improve the prognosis of acute trauma patients. The research results are similar. Multidisciplinary collaborative chain management mode can effectively utilize hospital emergency resources and can improve the emergency efficiency by building an integrated chain scraping glass mode with multidisciplinary participation, special personnel and post, and information sharing. Shortening the emergency time, the emergency process has the characteristics of high efficiency, high cohesion, high cooperation, high standardization, and high order to ensure that patients receive effective treatment as soon as possible to improve their prognosis of patients.

In conclusion, the multidisciplinary collaborative chain management model can shorten the emergency time of acute trauma patients, reduce the risk of adverse events, improve the quality of emergency care, and can improve the prognosis of the patients. The shortcoming of this study lies in that the included sample size is only 100 cases, and the sample size can be expanded in the future to further verify the results of this study.

## Figures and Tables

**Table 1:** Comparison of first aid efficiency between two groups ($\bar{x}$ ± *s*, min).

Groups	Emergency triage time	Time-consuming assessment	Time-consuming preparation of auxiliary equipment/devices	Time from admission to multidisciplinary consultation	From admission to receiving effective treatment
Chain group (*n* = 58)	4.81 ± 0.78	5.24 ± 1.69	5.72 ± 1.78	7.91 ± 2.52	27.52 ± 3.51
Regular group (*n* = 42)	6.02 ± 1.42	6.48 ± 1.76	6.90 ± 2.25	15.40 ± 3.15	35.76 ± 2.51
*t*	5.461	3.548	2.924	13.218	13.001
*P*	<0.001	0.001	0.004	<0.001	<0.001

**Table 2:** Comparison of the incidence of adverse events between the two groups (*n* (%)).

Groups	Doctor-patient disputes	Misdiagnosed	Missed diagnosis
Chain group (*n* = 58)	2 (3.45)	0 (0.00)	1 (1.72)
Regular group (*n* = 42)	6 (14.29)	3 (7.14)	5 (11.90)
*χ* ^2^	3.848	4.228	4.432
*P*	0.049	0.040	0.035

**Table 3:** Comparison of prognosis between two groups.

Groups	MODS (*n* (%))	ARDS (*n* (%))	Success rate of treatment (*n* (%))	Hospitalization period ($\bar{x}$ ± *s*, d)
Chain group (*n* = 58)	5 (8.62)	9 (15.52)	57 (98.28)	25.69 ± 6.81
Regular group (*n* = 42)	10 (23.81)	14 (33.33)	37 (88.10)	27.02 ± 7.85
*t/χ* ^2^	4.364	4.322	4.432	0.907
*P*	0.037	0.038	0.035	0.367

**Table 4:** Comparison of nursing quality between two groups ($\bar{x}$ ± *s*, points).

Groups	System construction	Emergency management	Safety management
Chain group (*n* = 58)	41.76 ± 2.84	92.26 ± 9.56	55.22 ± 5.42
Regular group (*n* = 42)	37.81 ± 4.92	88.12 ± 10.30	51.33 ± 8.03
*t*	5.065	2.069	2.893
*P*	<0.001	0.042	0.005

## Data Availability

The data can be obtained from the author upon reasonable request.
